# Operation for Enlarged Patellar Bursa

**Published:** 1843-03

**Authors:** 


*Operation for enlarged Patellar Bursa.*—Dr. Hargrave, of Dublin, performed the following operation on a healthy housemaid, twenty-one years of age, admitted into the city of Dublin Hospital, with enlarged patellar bursa.

June 8th, 1841.—"An incision to the extent of one-eighth of an inch, was made along the outer margin of the tumour; then a very small bistoury was introduced obliquely into the cyst, at such a distance from the superficial cutaneous incision as prevented the escape of the fluid.

"The sac was then cut in several places, chiefly on the anterior surface, and the instrument withdrawn, all the fluid having been evacuated.

"A small compress was then applied, and several strips of adhesive plaster, and a roller which extended from the toes to the knee.

"A splint was also applied, which extended from the middle of the back part of the thigh to the same point of the leg.

"10th. Dressings were removed; considerable diminution in size of swelling.

"Strips of adhesive plaster were again applied nearly in the same way as that recommended by Baynton.—No constitutional disturbance.

"14th. Strips quite loose. A strong evidence of subsidence of swelling.

"17th. Natural appearances of the joint nearly restored.

"Discharged at her own request, but strictly cautioned against returning to her usual employment for some time.

"If the incision, or rather punctures into the sac," Dr. Hargrave says, "be made with care, the internal surface of the cyst then cautiously scored after it, the fluid evacuated by firm pressure, so as to prevent the ingress of air into the cavity, no danger need be apprehended of unpleasant effects succeeding to this measure." "The only instances, he adds, "in which the subcutaneous incision might fail, are those where the sac is much thickened, its interior loculated, and the cells filled with a thick gelatiniform substance: still in such instances, it is a means which should be kept in view." *Am. Jour. Med. Sci.* from *Dublin Med. Press*, Oct. 26, 1842.

